# What is needed for improved uptake and adoption of digital aftercare programs by cancer survivors: a mixed methods study applying the COM-B model

**DOI:** 10.1007/s11764-024-01635-x

**Published:** 2024-07-04

**Authors:** Liza van Deursen, Rosalie van der Vaart, Niels H. Chavannes, Jiska J. Aardoom

**Affiliations:** 1 Department of National Health and Health Care, Center for Public Health, Health Care and Society, National Institute for Public Health and the Environment, Antonie Van Leeuwenhoeklaan 9, 3721 MA Bilthoven, the Netherlands; 2 National eHealth Living Lab, Leiden, the Netherlands; 3 Department of Public Health and Primary Care, Leiden University Medical Center, Leiden, the Netherlands; 4 Centre of Expertise Health Innovation, The Hague University of Applied Sciences, The Hague, the Netherlands

**Keywords:** e-health, Digital, Cancer, Oncology, Survivorship, COM-B, Behaviour, Quality of care, Psycho-oncology, Follow-up

## Abstract

**Introduction:**

Cancer survivors face physical, lifestyle, psychological, and psychosocial challenges. Despite the availability of aftercare services, survivors still have unmet needs. Digital aftercare programs may offer support, but their use is limited. This study aimed to examine what is needed to improve uptake and adoption of these programs. Additionally, it explored sociodemographic and clinical variables that may influence these needs.

**Methods:**

A mixed-methods approach was used, involving qualitative interviews and a questionnaire. The research was guided by the COM-B model of behaviour, which considers capability, opportunity, and motivation crucial for behaviour. Qualitative analysis was performed using the framework method. Statistical analyses involved descriptive statistics and regression analysis.

**Results:**

Fourteen cancer survivors were interviewed, and 213 participants completed the questionnaire. Findings indicated that most respondents had a positive or neutral attitude towards digital aftercare programs, believing these could address their cancer-related challenges. Still, only a small percentage had experience with them, and most were unaware of their existence. Many expressed a desire to be informed about them. Some were uncertain about their effectiveness. Others were concerned about a lack of reimbursement. No significant influence of the sociodemographic and clinical variables was found.

**Conclusion:**

Cancer survivors are generally positive about digital aftercare programs but are often unaware of their availability. Raising awareness, clarifying their value, and providing support and reimbursement could enhance uptake and adoption.

**Implications for Cancer Survivors:**

The current insights can help improve participation in digital aftercare programs, ultimately fostering health, well-being, and quality of life of cancer survivors.

**Supplementary Information:**

The online version contains supplementary material available at 10.1007/s11764-024-01635-x.

## Introduction

Cancer diagnoses have been on the rise in recent years. In the Netherlands, the number of patients receiving a cancer diagnosis has increased from 74,500 20 years ago to 124,100 in 2022 [1]. Worldwide, there is a 20% chance of developing cancer before the age of 75 [2]. Approximately 70% of adult male cancer patients and 66% of adult female cancer patients survive for at least 5 years after diagnosis [3]. Cancer survivors often experience physical, lifestyle, psychological, and psychosocial challenges after treatment. Examples of these challenges are fatigue, fear of recurrence, cognitive limitations, sexual dysfunction [4–9], and community reintegration problems, which include cancer-related financial and employment issues and issues in relating to friends and family members [10–12]. These challenges vary depending on the type of cancer and treatment and can persist long after treatment completion [13–15].

Previous research showed that 63% of cancer survivors have unmet needs after treatment. These needs are mainly related to emotional and social support, managing side effects, coping with the fear of recurrence, accessing up-to-date information, work support, and smoking cessation support [13]. As a result, addressing the needs of cancer survivors extends beyond the realm of medical care, requiring a broader commitment at the societal level.

The Health Council defines aftercare as an essential part of individual patient care after cancer treatment [16], which includes providing information and guidance, addressing complaints and symptoms, assessing direct or late effects (i.e. those consequences that do not yet exist, or at least do not present complaints at the end of treatment) of disease and treatment, and attention to social consequences [16]. Cancer survivors’ health, well-being, and quality of life benefit significantly from proper aftercare [17–19]. For example, nurse-led survivorship care has been shown to have positive patient-reported outcomes in areas such as cognitive and social functioning and fatigue [20].

In Dutch hospitals, cancer care regularly focuses mainly on the medical treatment provided by specialists, with little emphasis on psycho-social aftercare [21]. Additionally, general practitioners and specialist nurses often face difficulties in delivering aftercare due to time, resources, and knowledge constraints [10]. Other healthcare providers (HCPs), such as paramedics, psychologists, and informal caregivers from cancer meeting centers, can offer support on psychosocial issues. However, a large proportion of cancer survivors still do not receive appropriate care and support aimed at dealing with the (late) consequences of cancer (treatment) [22].

Digital self-management programs can be a helpful and accessible way to provide aftercare. Typically accessed through web or mobile applications, these programs cater to individual needs. For instance, a digital aftercare program may begin with users logging into a website and completing a questionnaire detailing their symptoms and challenges. Subsequently, a personalized program is composed based on these responses. This tailored approach encompasses various elements such as informational resources, expert advice, shared experiences from fellow cancer survivors, and interactive assignments on topics such as fatigue, fear of cancer recurrence, and lifestyle [23–33]. For instance, one assignment might involve maintaining an activity diary to discern which activities drain energy and which replenish it for the individual.

Digital self-management programs can alleviate the strain on care for cancer survivors and help survivors develop self-management skills. A recent systematic review in the Netherlands found that several initiatives have been developed and scientifically examined, demonstrating promising results [34]. These initiatives have shown to be effective in, for example, improving physical activity and sleep quality, as well as reducing depressive symptoms [34–36].

Despite the benefits of digital interventions that address cancer-related issues, their impact is often limited due to their restricted reach [37, 38]. Studies have shown that the uptake and adoption of such interventions is hindered by several factors, including limited perceived usefulness and usability, technical difficulties, and lack of time, motivation, and familiarity among (potential) users [39–42].

To better understand the factors that affect the use of digital aftercare programs in cancer care, the current study aims to answer the following research question: What do cancer survivors need for improved uptake and adoption of digital aftercare programs? To examine this, the Capability, Opportunity, and Motivation Behaviour (COM-B) model is used as a framework [43]. This model is a widely used behavioural change model in digital health intervention research [44, 45]. According to this model, individuals can only engage in a specific behaviour, such as using digital aftercare programs, if they have the capability, opportunity, and motivation to do so [46].

Research has shown that certain sociodemographic factors, such as age, income, and education level, can affect the use of digital applications. Specifically, individuals who are older, or have lower income or educational levels, tend to use these applications less often [47–49]. This indicates that the extent to which people use digital applications varies. Additionally, research has indicated that clinical factors, such as cancer type and time elapsed since treatment, can influence the challenges experienced after treatment [13–15, 50], which may affect the need for (digital) aftercare. However, it remains unclear whether there are any variations in what is required to encourage the uptake and adoption of online aftercare programs, particularly for cancer survivors. Therefore, the second research question aims to address this gap: Are there any sociodemographic or clinical variables that influence cancer survivors’ needs regarding the uptake and adoption of digital aftercare programs?

There has been little research conducted on the uptake of digital aftercare interventions for cancer survivors. The current study contributes to this field of research and takes a new approach by being the first to use the COM-B behavioural model to study the factors that influence survivors’ participation in digital aftercare programs. Additionally, this study contributes to the existing literature by exploring bottom-up the needs of cancer survivors regarding the uptake and adoption of digital programs, followed by surveying a broad (more representative) group about these needs. This comprehensive approach enhances the reliability of findings. Finally, this study examines the specific needs of diverse groups of cancer survivors, considering sociodemographic and clinical factors. Together, this presents a thorough overview of needs, to understand and improve the utilization of digital aftercare programs in specific populations.

## Methods

### Study design

The study employed a mixed-methods design, using semi-structured interviews and a self-constructed questionnaire to gain a comprehensive understanding of the subject through methodological triangulation [51]. The first research question was answered by conducting the interviews to identify themes and gain context, followed by the questionnaire to verify the consistency of information among a larger and more diverse group of cancer survivors. To enhance accessibility and convenience, the interviews were conducted online via videoconference, and the questionnaire was distributed digitally. This approach may introduce a response bias, as individuals who are more likely to engage with online resources are also the ones providing data on their needs for digital programs. However, considering that this demographic constitutes the primary target group, this method was selected to facilitate their participation as much as possible.

For the second research question, exploratory analyses were performed on the questionnaire data to determine whether sociodemographic or clinical variables influenced the needs of survivors for the uptake and adoption of digital aftercare programs.

### Conceptual framework

The qualitative interviews, the questionnaire, and the data analyses were based on the COM-B model of behaviour [43], a widely used approach to understanding behaviour and behavioural change in the context of health. The model is instrumental in designing behavioural interventions or approaches that effectively target specific factors influencing behaviour. The COM-B model identifies three key factors for a behaviour to occur: capability, opportunity, and motivation [52]. Capability refers to an individual’s psychological and physical ability (i.e. knowledge, skills, and abilities) to engage in the behaviour. Opportunity encompasses external factors that enable or prompt the behaviour, such as social and physical circumstances. Motivation encompasses conscious and unconscious processes that drive behaviour, including emotion and impulse [52]. The interviews and questionnaire included questions to measure cancer survivors’ capability, opportunity, and motivation to utilize digital aftercare programs. Furthermore, the data were analyzed using the COM-B model as a framework for the coding scheme.

### Sampling and recruitment

Participants were recruited for the interviews through Kanker.nl (Cancer.nl), a Dutch national online platform for cancer survivors and their relatives [53]. Relevant users on this platform who had given permission to be approached for scientific research received an email invitation. The invitation contained a sign-up link that directed them to a short online questionnaire to determine their eligibility for the study. Participants were eligible for the study if they had been diagnosed with cancer and completed treatment within the past 5 years and were proficient in Dutch. In addition, they had to report a need for support or information during the aftercare phase, as the study aimed to investigate the motivations and factors specifically related to the adoption and uptake of digital aftercare programs, rather than broader considerations regarding the need for aftercare itself. As a token of appreciation for their participation, respondents received a €25 gift voucher, which was communicated during recruitment.

A purposive sampling method [54] was used to select participants from the list of applicants to ensure diversity in demographics (age, gender, and education), cancer type, and duration since treatment completion. The sample size was not predetermined, and interviews were conducted until data saturation was reached. Out of 46 applicants who met the eligibility criteria, 16 were invited for an interview. However, two of them withdrew from participation, which resulted in a total of 14 participants. All participants were fully informed about the objectives and characteristics of the study and provided written consent.

To gather respondents for the questionnaire, invitations containing the link to the questionnaire were emailed to the same group of Kanker.nl users who were approached for interviews. Respondents were also recruited through the LinkedIn pages of the researchers and their organizations and by posting invitations to Dutch social media (i.e., Facebook) groups for cancer survivors. Three €50 gift vouchers were raffled among the respondents, as communicated in the recruitment messages.

Before starting the questionnaire, all respondents received written information detailing the study’s objectives and procedures. Respondents were required to confirm that they had read, understood, and agreed to the goals and procedures of the study, as well as their rights as respondents.

### Data collection

The online interviews were conducted in April and May 2023 in Dutch by two female researchers with a background in psychology and trained in interview techniques (author LvD, MSc and RvdV, PhD). A semi-structured protocol, which consisted of open-ended questions and probes, was used during the interviews. This allowed the interviewers to adjust the order of questions or clarify them when necessary [55]. The protocol was pilot tested with a cancer survivor before the study began, resulting in minor adjustments. All components of the COM-B model were covered in the protocol. The questions were based on sample questions developed by the University College London Centre for Behaviour Change [56], tailored to the context and method of this study. Some examples of the resulting questions were as follows: 1. Have you ever used online aftercare programs? If so, what did you like, and what did you miss? (behaviour); 2. Can you tell us how familiar you were with online aftercare programs before this conversation? What do you know about them? (opportunity); 3. What advantages do you perceive to gain from using online aftercare programs? (motivation); 4. How easy or difficult do you think using online aftercare programs would be for you? What could make it easier for you? (capability). The complete interview guide can be found in Supplementary File 2.

At the start of the interview, each participant was presented with a hypothetical example of a digital aftercare program based on existing programs. The design of the hypothetical program included a personalized questionnaire to determine which modules would be most relevant to the participant, followed by several modules providing information, videos from cancer survivors and HCPs, and assignments. The program would address topics such as fear of cancer recurrence, physical activity, and fatigue. The interviews, which were video recorded and transcribed verbatim, lasted approximately 1 h. Data saturation was achieved for the identified themes after 14 interviews.

The questionnaire was launched in July 2023 and could be filled out by respondents until September 2023. The questionnaire contained 49 questions that were based on the COM-B model and the interview results. The interview results were used to select and operationalize the relevant themes related to the COM-B concepts to be included in the questionnaire. The questionnaire started with an assessment of demographics. Then, it continued with questions about each COM-B category, addressing current usage, motivation, capability, and opportunity for the uptake and adoption of digital aftercare programs. A video was created to explain the concept of digital aftercare programs, which respondents were instructed to watch before completing the questionnaire. The video was based on the hypothetical example of a digital aftercare program given during the interviews. The questionnaire was hosted on Formdesk, which is a web-based survey platform [57]. It took about 15 min to complete. The questionnaire can be found in Supplementary File 3.

### Data analysis

The interviews were analyzed using MAXQDA 2022 software [58]. The analysis process was deductive, using the conceptual categories from the COM-B model as a framework [46], and inductive, adding new categories deriving from the data. The framework method [59] was used for data analysis, which is a qualitative content analysis approach adaptable for generating themes. Two researchers, LvD and RvdV, independently coded the first three interviews, after which the researchers collaborated to create a common coding framework for all the data. The coding framework was refined through continued collaboration and discussions to resolve discrepancies during the coding of all subsequent interviews. A framework matrix was created to summarize the data from each interview, and finally, the data was interpreted. The Consolidated Criteria for Reporting Qualitative Research (COREQ) checklist was used to ensure research quality (Supplementary File 4) [60]. Illustrative quotes were translated into English, and the following information was added: participant number, gender, and age in years.

The IBM SPSS Statistics version 28 was used to analyze the data of the questionnaire [61]. Descriptive statistics were used to summarize the questionnaire items (Supplementary File 5). Exploratory regression analyses were conducted to examine whether sociodemographic or clinical variables influenced the needs of cancer survivors for the uptake and adoption of digital aftercare programs (Supplementary File 6). Four sociodemographic variables were analyzed: age, educational level, income, and marital status, as well as two clinical variables: type of cancer and duration since treatment completion. The exploratory analyses were conducted on four questions from the questionnaire designed to measure the main components of the COM-B model. For questions requiring respondents to choose one or multiple options, only those selected by at least 15% of the respondents were included in the analyses. The data was analyzed using binary logistic and ordinal regression analyses, as appropriate. Due to the large number of tests conducted, the Benjamin Hochberg FDR correction [62] was applied to correct for multiple testing.

## Results

### Characteristics of interview participants and questionnaire respondents

Interviews were held with fourteen participants: seven men and seven women. Six individuals completed secondary vocational education (42.9%), three completed post-secondary vocational education (21.4%), and five completed higher professional or academic education (35.7%). Most participants were diagnosed with either breast (*n* = 4; 28.6%), skin (*n* = 3; 21.4%), or bladder (*n* = 2; 14.3%) cancer. The majority finished treatment either less than 1 year ago (*n* = 3; 21.4%), 1 to 2 years ago (*n* = 5; 35.7%), or 3 to 4 years ago (*n* = 4; 28.6%). More information on interview participants’ characteristics can be found in Supplementary File 7.

In the study, a total of 282 individuals responded to the questionnaire. Out of these, 69 individuals were excluded from the analysis as they did not meet the inclusion criteria for participation. This was because they stated they did not require assistance with their challenges and complaints. The remaining 213 individuals’ data were used for the analyses. Table 1 provides an overview of the characteristics of these respondents. For more information on the respondents’ characteristics, please refer to Supplementary File 5.

**Table 1:** Questionnaire respondents’ characteristics

Characteristics	Total (*N* = 213)	
	***n***	**%**
Gender		
Male	70	32.9
Female	141	66.2
Non-binary	2	0.9
Age		
Mean (*SD, min–max*)	71 (10.6, 23–94)	
Marital status^1^		
With partner	154	72.3
Without partner	58	27.2
Educational level		
Secondary (vocational) education	43	20.2
Post-secondary vocational education	52	24.4
Higher professional education or academic education	118	55.4
Difficulty making ends meet from household income in the past twelve months^2^		
Yes	48	22.5
No	164	77.0
Cancer type^3^		
Breast cancer	51	23.9
Colorectal cancer	22	10.3
Bladder cancer	20	9.4
Prostate cancer	16	7.5
Throat or laryngeal cancer	13	6.1
Esophageal cancer	11	5.2
Ovarian cancer	8	3.8
Skin cancer	8	3.8
Multiple types of cancer	8	3.8
Uterine or cervical cancer	7	3.3
Lymph node cancer	7	3.3
Lung cancer	6	2.8
Brain tumour	5	2.3
Other types	31	14.5
Time since treatment completion^4^		
Currently undergoing treatment	34	16.0
Less than one year ago	46	21.6
One to two years ago	53	24.9
Three years or longer ago	80	37.6

^1^With partner: married or registered partnership (*n* = 123, 57.7%); in a relationship (not married or in a registered partnership (*n* = 31, 14.6%). Without partner: single (*n* = 29, 13.6%); divorced (*n* = 15, 7.0%); widow(er) (*n* = 14, 6.4%). Other (*n* = 1, 0.5%)

^2^Yes: significant difficulty (*n* = 10, 4.7%); some difficulty (*n* = 38, 17.8%). No: no difficulty, but I need to watch my expenses (*n* = 78, 36.6%); no difficulty (*n* = 86, 40.4%). I would rather not say (*n* = 1, 0.5%)

^3,4^An overview of the other types of cancer and data on the duration since treatment is completed can be found in Supplementary File 5

The results of the qualitative analyses of the interviews and the descriptive analyses of the questionnaire data are presented below. Additional details on these results can be found in Supplementary File 5.

### Behaviour—experiences with the (digital) aftercare interventions

All interview participants experienced challenges and complaints after treatment completion, as this was an inclusion criterion to participate in the interviews. They frequently mentioned fatigue, fear of recurrence, and difficulty in processing their experiences with the disease and the treatment. Pain complaints, and challenges related to nutrition and exercise were also mentioned.

Participants were asked about their current in-person and digital aftercare usage. A small group of participants had in-person contact with HCPs such as physiotherapists, psychologists, or dieticians. Some sought information from patient associations or walk-in centers. Half of those interviewed used online opportunities to connect with peers, for example, through social media. No interviewee utilized stand-alone digital aftercare programs as outlined by the researchers in the hypothetical example. Two individuals participated in blended aftercare programs that combined online conversations or modules with in-person consultations with a psychologist.

*“An online aftercare program would definitely be something that appeals to me, at least. There was a point when I really felt the need for it and even looked for it, but I didn't find anything suitable” *(Interview 3, woman, 62 years old). 

The questionnaire respondents reported facing various challenges and complaints after treatment. The ones most commonly listed were fatigue (*n* = 162; 76.1%), fear of cancer recurrence (*n* = 119; 55.9%), concentration problems (*n* = 102; 47.9%), and pain (*n* = 100; 46.9%). Almost all respondents agreed (completely) with the following statement: “I think it is important to address my complaints or challenges to alleviate the resulting stress” (*n* = 196; 92%). Participants sought help from various sources, which included visiting a general practitioner (*n* = 108; 50.7%), a physiotherapist (*n* = 130; 61%), or a psychologist (*n* = 91, 42.7%). In addition, over two-thirds of the participants searched for information online (*n* = 143; 67.1%). Some sought digital peer support, for example, via Kanker.nl (*n* = 96; 45.1%). Only a small percentage of respondents had used digital aftercare programs before (*n* = 21; 9.9%).

### Capability—knowledge, skills, and ability to use digital programs

Digital aftercare programs were largely unknown among the interview participants; only one person was familiar with them. Numerous participants have expressed their desire to be informed about the available programs. After completing their treatment, a substantial number of participants felt unsupported and left to fend for themselves. They believed that digital aftercare programs could have been helpful during this phase.

“*Yes, awareness is the most important thing. That people know there is more help available than just the hospital”* (Interview 14, woman, 56 years old).

Participants had varying opinions on who should inform them about digital aftercare programs. While most participants believed that the hospital should facilitate this, specifically the doctor or nurse, some preferred to be informed through their general practitioner or notified through social media. Most participants did not have a specific preference about the timing of when they should receive this information during their treatment process. Half of the participants expressed confidence in their ability to use digital aftercare programs, believing that they could easily navigate them. Others stated that they could use the programs but emphasized the need for accessible and user-friendly designs. A small group of participants felt that they lacked sufficient digital skills and needed to improve them to use the programs effectively. Participants suggested a clear explanation of the program, IT help desks and support websites to support those with less digital literacy skills. Furthermore, they mentioned that senior citizens’ associations, domiciliary care, libraries, and individuals’ social networks could serve as potential support providers.

“*I spoke with a 74-year-old woman with breast cancer, and she feels completely abandoned. But she also doesn't seek help herself because she's not from the generation that uses computers”* (Interview 7, woman, 49 years old).

The questionnaire results showed as well that most respondents (*n* = 180; 84.5%) were unfamiliar with digital aftercare programs, as explained in the video before filling out the questionnaire. Of the 33 respondents (15.5%) who had heard of them, most were informed by their oncological nurse (*n* = 8; 24.2%) or the Kanker.nl website (*n* = 11; 33.3%). Of the 21 respondents (9.9%) with prior experience using digital aftercare programs, 47.6% (*n* = 10) (completely) agreed that they addressed their challenges, 38.1% (*n* = 8) had no opinion, and 14.3% (*n* = 3) (completely) disagreed.

Most respondents would have liked to have been informed about the existence of digital aftercare programs (*n* = 205; 96.3%), which should be done preferably during (*n* = 93; 43.7%) or immediately after completing treatment (*n* = 115; 54.0%). Respondents preferred to hear about it from HCPs such as their medical specialist (*n* = 131; 61.5%), (oncological) nurse (*n* = 127; 59.6%), or general practitioner (*n* = 99; 46.5%). Other frequently listed sources were the website Kanker.nl (*n* = 84; 39.4%) and patient associations (*n* = 52; 24.4%).

Most respondents agreed with the statement that they possessed sufficient digital skills to use digital aftercare programs (*n* = 189; 88.7%). However, almost half of them (*n* = 100; 46.9%) still expressed the need for further assistance, such as through a digital helpdesk (*n* = 60; 28.2%), a phone number to call (*n* = 35; 16.4%), or a program tutorial (*n* = 30; 14.1%). About 40% of the respondents (*n* = 85; 39.9%) reported they did not require any help using the programs, and 13.1% did not know (*n* = 28). Apart from digital skills, other factors that could hinder respondents from using digital aftercare programs were a lack of energy (*n* = 58; 27.2%) and concentration (*n* = 48; 22.5%).

### Opportunity—external factors that enable or prompt the use of digital programs

Related costs and reimbursement were external factors influencing the opportunity to use the programs among interview participants. Participants expressed varying opinions regarding their willingness to pay for digital aftercare programs. Many could pay for such programs but would only be willing if they were proven effective. On the other hand, some participants stated that they were not willing to spend money on digital aftercare programs. Some reasons for this included limited funds and the belief that health insurance should cover aftercare. Some participants mentioned that offering programs for free would make them more accessible.

“*I do think it should be covered. Because it's not for everyone, it's for specific groups. There are already so many cutbacks, and you already have to pay for so much yourself. I think these kinds of things should just be taken care of by health insurance*” (Interview 10, man, 42 years old).

The participants did not experience any major obstacles due to the absence of information and communication technology (ICT) resources, as most of them possessed the necessary equipment. They also expressed that they would be able to manage their time effectively to use the digital programs. Furthermore, the participants reported that their immediate social environment supported their recovery. However, their support would not be a decisive factor in the participants’ decision to use digital aftercare programs.

According to the questionnaire results, several external factors may hinder respondents from using digital aftercare programs. A crucial factor was the payment for digital aftercare programs. Some participants indicated they had little money to purchase a digital aftercare program (*n* = 26; 12.2%). Most respondents did not want to pay anything for the use of the program (*n* = 166; 77.9%). Of those willing to pay (*n* = 47; 22.1%), the average amount they wanted to pay would be 48.8 euros (SD = 36.6; range = 10–150). For 60.1% of respondents (*n* = 128), it was crucial that their health insurance entirely financed the program. Another relevant factor influencing the use of digital aftercare programs was having concerns about privacy (*n* = 32; 15.0%). However, about a quarter of the respondents (*n* = 56; 26.3%) did not believe any factor could prevent them from using a digital aftercare program.

In terms of the role of the social environment, respondents generally expected their social environments, such as friends, family, and colleagues, to have a (very) positive (*n* = 99; 46.5%) or neutral (*n* = 68; 31.9%) attitude towards digital aftercare programs. Part of the respondents did not know the opinion of their social environment on the matter (*n* = 43; 20.2%). Most respondents (completely) disagreed with the statement: “The opinion of the people in my surroundings would influence my decision to use digital aftercare programs” (*n* = 144; 67.6%), while 26.8% (*n* = 57) neither agreed nor disagreed, and 35.7% (*n* = 76) (completely) agreed. Only a few respondents had someone in their social surroundings using digital aftercare programs (*n* = 12; 5.6%). Most respondents believed that their HCPs would have a (very) positive (*n* = 123; 57.8%) or neutral (*n* = 43; 20.2%) attitude towards digital aftercare programs. Some respondents indicated they did not know their HCPs’ opinions (*n* = 42; 19.7%). The respondents held different beliefs regarding whether their healthcare providers’ opinions would affect their decision to use digital aftercare programs. Among them, 37.6% (*n* = 80) completely agreed with the statement “The opinion of my healthcare providers would influence my decision to use digital aftercare programs”, while 30.5% (*n* = 65) completely disagreed and 25.4% (*n* = 54) neither agreed nor disagreed.

### Motivation—(un)conscious processes that drive the use of digital programs

Many interview participants expressed their willingness to use digital aftercare programs to address their challenges. They believed that these programs could offer a sense of validation for individuals who feel misunderstood or unsupported by their social environment when they encounter difficulties after treatment has ended.

“*And also with those fatigue complaints, if there is a good way to work on that, then I would definitely make use of it*” (Interview 3, woman, 62 years old).

However, some participants expressed uncertainty or skepticism towards digital aftercare and believed that in-person care was superior. During the study, the participants shared their thoughts on the pros and cons of digital aftercare. They mentioned that digital aftercare had several benefits such as being convenient, accessible, and flexible. It also eliminates waiting lists, saves time and costs, can prevent further care, and allows patients to pause the program or revisit information.

“*I can set it aside for a moment and think about it. So that's easier than when you're talking with someone, because when you're talking with someone, you want to be able to give an immediate answer, and that just doesn't always work. Sometimes I just can't come up with things*” (Interview 14, woman, 56 years old).

However, some participants felt that it could be impersonal and requires a lot of self-discipline to continue. While a few preferred in-person support, most believed that the advantages of digital aftercare outweighed the disadvantages. A few were willing to consider digital aftercare but distrusted commercial programs.

Concerning the program’s content, participants desired personalized programs tailored to their needs. They would prefer programs that offer information, tips, and advice on how to deal with various issues and situations. Some participants would like to read about the experiences of others, while others would appreciate direct contact with fellow sufferers. Assignments would be helpful to some, and others would appreciate references within the program for further information. Almost all participants were motivated to use online programs to reduce the impact of their challenges and complaints on their daily lives. Participants suggested receiving regular reminders and feedback and adjusting the program based on their results to stay motivated. Although not typically part of stand-alone digital aftercare programs, almost half the participants wished to have contact with an HCP or an experienced expert in addition to the program.

“*When it comes to fatigue, it's nice to have tips. For example, knowing which exercises you should do, how long you should do them, and perhaps something related to diet. Like saying: well, it's best not to eat too much of this, but make sure you get plenty of fruits and vegetables*” (Interview 2, man, 62 years old).

Regarding the questionnaire results, it was observed that most respondents had a positive (*n* = 133; 62.4%) or neutral (*n* = 69; 32.4%) attitude towards digital aftercare programs. To the statement: “Digital aftercare programs can help me with the complaints or challenges I am experiencing due to cancer or cancer treatment”, most respondents (completely) agreed (*n* = 130; 61.0%) or neither agreed nor disagreed (*n* = 50; 23.5%). When reflecting on their motivation to use digital programs, most respondents believed that the benefits of using a digital aftercare program would outweigh the disadvantages (*n* = 114; 53.5%), while some did not know (*n* = 76; 35.7%), and only a few did not think the benefits would outweigh the advantages (*n* = 23; 10.8%). The most frequently selected advantages (from a list of options) of digital aftercare programs were being in control of when to use it (*n* = 179; 79.8%), the option of re-reading information (*n* = 132; 62.0%), being able to instantly (without a waiting list) (*n* = 80; 37.6%) and independently (*n* = 75; 35.2%) work on challenges and complaints, and receiving support in the phase after treatment completion (*n* = 68; 31.9%). Out of the options provided, the most common selected drawbacks were the inability to have personal contact (*n* = 133; 62.4%), the absence of opportunity to ask questions (*n* = 106; 49.8%), and the fact that it takes effort to continue using the program (*n* = 76; 35.7%). Out of all respondents, 30.0% expressed doubts about the effectiveness of digital aftercare programs (n=64), while 13.6% had concerns about their reliability (n=29).

Regarding staying motivated to use digital programs for a more extended period, part of the respondents would find it (very) difficult to consistently use a digital aftercare program a few times a week (*n* = 42, 20.6%). A larger group neither finds it hard nor easy (*n* = 78; 36.6%), while other respondents find it (very) easy (*n* = 70; 32.9%), and 9.9% did not know (*n* = 21). The questionnaire results indicate that the factors that would encourage people to use these programs frequently include personalized tailoring of the program to their situation (*n* = 134; 62.9%), providing insight into the duration and the completed parts of the program (*n* = 124; 58.2%), and providing feedback based on their activities (*n* = 91; 42.7%). In addition, respondents expressed interest in having digital contact with an HCP or coach (*n* = 87; 40.8%), and receiving regular reminders (*n* = 73; 34.3%).

### Exploratory analyses regarding possible influencing variables

Supplementary file 6 contains the output of the exploratory analyses regarding possible influencing sociodemographic and clinical variables on four questions representing the main components of the COM-B model. The first question assessed respondents’ familiarity with digital aftercare programs. Binary logistic regression analysis revealed no significant associations between the sociodemographic and clinical variables and respondents’ responses to this question. The second question explored possible differences in respondents’ preferences of how to be informed about digital aftercare programs. Separate binary logistic regression analyses were conducted for each answer option selected by at least 15% of respondents. Initially, the results showed significant associations between the variables Age and Type of Cancer, and the answer option “through the medical specialist” (*p* = 0.006; *p* = 0.004, respectively). A significant association was also found between the variable Educational level and the answer option “through the nurse” (*p* = 0.006). However, after applying the Benjamin Hochberg False Discovery Rate (FDR) correction, these three associations were no longer significant (*p* = 0.18; *p* = 0.18; *p* = 0.18 respectively). The third question investigated factors that could deter respondents from using digital aftercare programs. Initially, the results revealed significant associations between the variable Age and the answer option “Difficulty concentrating” (*p* = 0.029), and the variable Type of cancer and the answer option “no factors” (*p* = 0.02), but after applying the Benjamin Hochberg FDR correction, these associations lost their significance (*p* = 0.44; *p* = 0.40, respectively). The fourth question assessed respondents’ agreement with the statement, “I would like to address my complaints or challenges independently and online”. An ordinal regression analysis initially found one significant association for the variable Time Since Treatment Completed (*p* = 0.009). This association was no longer significant after applying the Benjamin Hochberg FDR correction (*p* = 0.20).

## Discussion

The primary research question of this mixed-methods study was the following: what is needed for improved uptake and adoption of digital aftercare programs by cancer survivors? The study’s findings suggest that cancer survivors are generally positive about using digital aftercare programs. They value the possibility to use these programs independently and on their own terms. They recognize the potential of such programs in addressing various challenges they face, such as fatigue, fear of recurrence, coping with illness, and pain complaints. Other studies have also found positive attitudes among cancer survivors towards digital aftercare programs. For instance, a study by Melhem et al. (2023) found that many cancer survivors are interested in using mobile applications to access cancer-related information during survivorship [47], while another study by Vogel et al. (2021) found that 68.7% of cancer survivors believed that an app would be an ideal complement to standard follow-up [63]. However, despite this positive attitude, the current study also found that usage of digital aftercare programs among cancer survivors is very low, which is consistent with previous research that found low adoption rates of mobile technologies among cancer survivors [64].

During the study, several key factors were identified that could potentially enhance the uptake and adoption of digital care programs among cancer survivors. Notably, it was observed that many survivors are often unaware of the existence of these programs, despite two-thirds of questionnaire participants indicating they searched for information on complaints and challenges related to their cancer or cancer treatment online. This finding aligns with previous research indicating that patients often lack knowledge about their e-health options, resulting in the underutilization of such programs [65]. The low discoverability of digital care programs underscores the need for improved visibility and accessibility to ensure that survivors can easily find these programs and benefit from them.

Survivors would appreciate being actively informed about the programs, preferably by their medical specialist or nurse. Although the social environment does not seem to influence survivors’ decision to use digital aftercare programs, healthcare professionals’ opinions about the programs are very important for some survivors. Therefore, it is important for healthcare professionals to inform their patients about the available programs, their effectiveness based on evidence, and the possible benefits that the programs can provide for the patient. It is crucial to understand the preconditions and needs of healthcare professionals to effectively perform their role as a referrer. Therefore, it is essential to determine if healthcare professionals are familiar with- and willing to recommend such programs. Incorporating information about online aftercare programs into HCP educational programs can be beneficial, enabling them to know the options, which ones have been proven effective, and which ones they can be confidently recommend.

Additionally, some survivors have doubts about the effectiveness of digital aftercare programs, and some considered traditional in-person care to be superior. These doubts may limit their willingness to use such programs and hinder their ability to benefit from them [66]. In these cases, a blended approach, combining online components through digital aftercare programs with face-to-face interaction with human care providers, could be an adequate solution [67]. This blended care approach is commonly used in e-health and could overcome the limitations of digital programs, although it introduces its own set of challenges [67–70]. In fact, our study found that many respondents would prefer a combination of a digital aftercare program with (digital) contact with a healthcare provider.

Furthermore, our study discovered that certain design elements of digital aftercare programs are vital to motivate survivors to use them consistently. Personalized programs that provide feedback on the progress made, by offering insights into their activities, were found to be more engaging for survivors. Therefore, tailoring information, advice, and support to the individual’s use and needs can enhance digital aftercare programs. Evidence has shown that tailored web-based interventions on health behaviours are more effective than non-tailored interventions in affecting health outcomes [71]. For example, a meta-analysis by Lustria et al. (2013) demonstrated the effectiveness of tailored web-based approaches to health interventions [72].

Finally, the availability of free digital aftercare programs positively impacts their uptake and adoption by cancer survivors. Currently, the provision of these programs varies. Some are offered by healthcare institutions, such as general practitioners, mental healthcare organizations, or hospitals. Others are provided for free by private organizations or patient organizations. Finally, some programs must be purchased by patients. Many survivors hesitate to pay for these programs out of pocket and would prefer their healthcare insurer to cover the costs. Thus, it is crucial to organize these programs so they are included in the reimbursed care package. To address the issues of availability and accessibility, a Dutch initiative called the Cancer.nl Appstore was recently launched [73, 74]. The AppStore, financed by the Dutch Cancer Society, is a central landing page via a reliable source. Currently, cancer survivors can receive a digital budget of one hundred euros via the website. This budget enables them to access interventions that have been labeled evidence-based and user-friendly based on the test method of the Dutch Public Health Service [75]. Also, healthcare professionals and other relevant parties could use the AppStore to easily refer patients to digital aftercare programs.

Another strategy that could be used to increase awareness of the possibilities of digital programs among cancer survivors is using public health campaigns via social media, which has been successfully done in the past to improve knowledge and attitudes towards cancer prevention [74]. Additionally, community-based outreach programs can be applied to reach cancer survivors in diverse populations [76]. Research is needed to examine whether these approaches encourage more survivors to uptake and adopt these programs.

The study’s second research question was whether certain sociodemographic and clinical variables influence the needs for the uptake and adoption of digital aftercare programs. However, based on the current exploratory analyses, there was insufficient data to demonstrate the possible influence of the studied variables. Therefore, additional analyses should be conducted using larger datasets to examine these and other variables to enhance the generalizability of the findings.

This study had several strengths, one of which was the use of a mixed-methods approach. This approach made it possible to cross-verify findings, increasing the overall validity and reliability of the results. Additionally, the use of the COM-B model provided a structured theory-based approach to understand the complex interplay of several factors. However, there were some limitations that need to be acknowledged [77]. First, the study relied on convenience samples and self-reported data, which may have led to a selection and response bias [78]. Additionally, since the interviews and the questionnaire were conducted and distributed digitally, individuals with digital skills are likely overrepresented. However, given the large group that needs additional support while relatively few make use of digital programs, it provides valuable insight to start with those who are already using the internet. They are the primary target for broader outreach with this type of intervention. Finally, the data was cross-sectional, which limits the ability to establish causal relationships and observe temporal changes [79].

## Conclusion

In this mixed-methods study, guided by the COM-B model, we integrated qualitative and quantitative approaches to gain valuable insights into cancer survivors’ views on what is needed for their improved uptake and adoption of digital aftercare programs. The study showed that cancer survivors are generally positive about using digital aftercare programs and recognize their numerous benefits. However, many survivors are unaware of the existence of these programs. For the uptake and adoption of digital aftercare programs, it is essential to raise awareness, clarify their value, and ensure that funding and support are available for survivors. The results of this study can be used to improve survivors’ access to and utilization of digital aftercare programs, which may ultimately foster post-treatment outcomes.

## Supplementary Information

Below is the link to the electronic supplementary material.

Supplementary file1 (DOCX 98 KB)

Supplementary file2 (DOCX 29 KB)

Supplementary file3 (DOCX 44 KB)

Supplementary file4 (DOCX 27 KB)

Supplementary file5 (DOCX 78 KB)

Supplementary file6 (DOCX 42 KB)

Supplementary file7 (DOCX 25 KB)

## Data Availability

No datasets were generated or analysed during the current study.
