# Supplementary figures and images for: What is needed for improved uptake and adoption of digital aftercare programs by cancer survivors: a mixed methods study applying the COM-B model
Source: J Cancer Surviv. 2024 Jul 4;20(1):323–35. doi: 10.1007/s11764-024-01635-x (PMC12906513; doi:10.1007/s11764-024-01635-x)

Supplementary File 1. Illustration of a digital aftercare program (in Dutch)


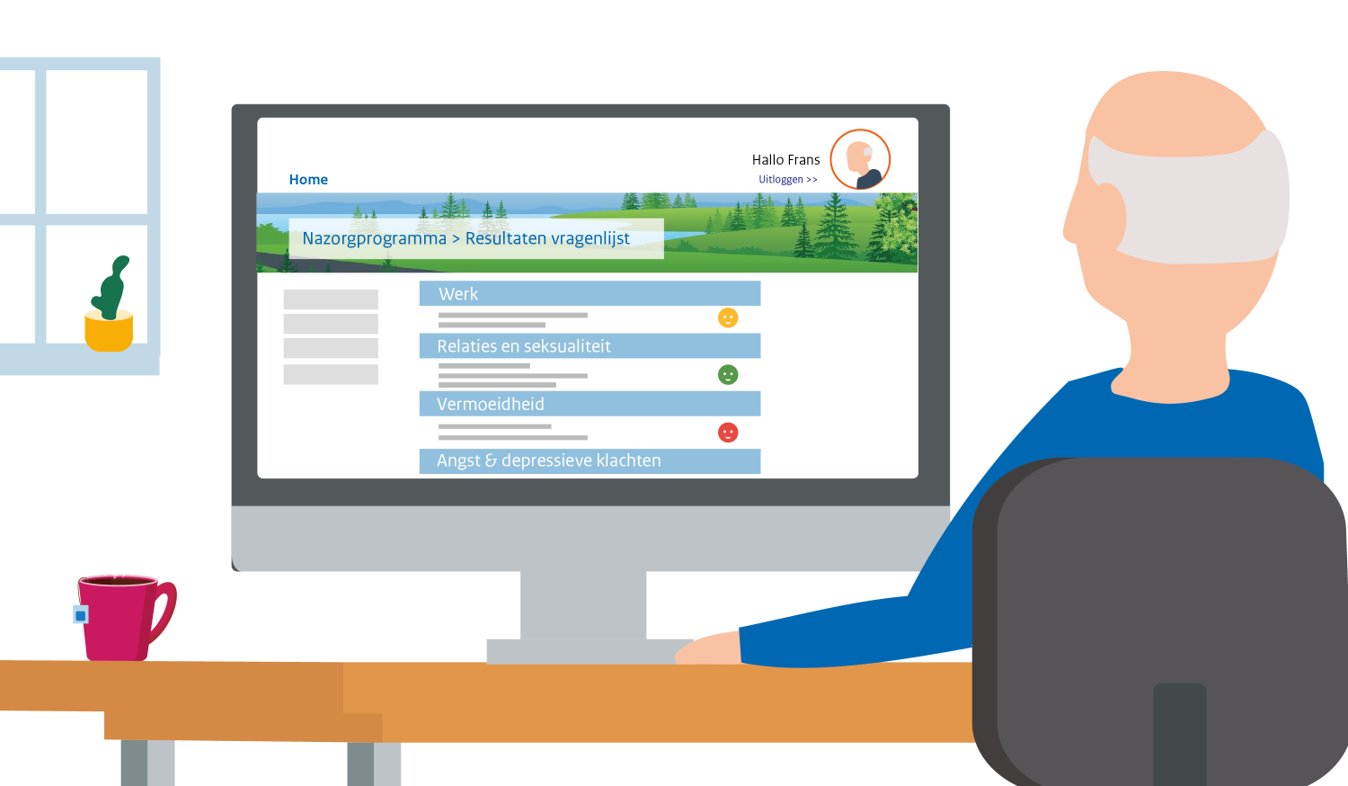

Supplement: Supplementary file 1 — Supplementary file1 (DOCX 98 KB) [file 11764_2024_1635_MOESM1_ESM.docx]
