# Supplementary material for: What is needed for improved uptake and adoption of digital aftercare programs by cancer survivors: a mixed methods study applying the COM-B model
Source: J Cancer Surviv. 2024 Jul 4;20(1):323–35. doi: 10.1007/s11764-024-01635-x (PMC12906513; doi:10.1007/s11764-024-01635-x)
Supplement: Supplementary file 2 — Supplementary file2 (DOCX 29 KB) [file 11764_2024_1635_MOESM2_ESM.docx]

Supplementary File 2. Interview guide [English translation]

**Introduction and Context**

[introduction slide]

First of all, thank you very much for agreeing to participate in this conversation. I would like to start this conversation by introducing myself and explaining the purpose and context of the research. After that, I would like to hear more about you.

My name is [name of the interviewer], and I work as a researcher for the E-health Monitor. The E-health Monitor is a collaborative project of the RIVM, NeLL, and Nivel, all organizations that conduct national research on healthcare. We started this project in 2021, commissioned by the Ministry of Health, Welfare, and Sport.

The goal of this project is to assess the use of digital healthcare and learn how digital healthcare can contribute to improving healthcare. By digital healthcare, we mean the use of ICT in healthcare, such as video calling or the use of a patient portal. In this conversation, we will focus on a specific form of digital healthcare, namely online aftercare, which I will discuss later.

It's important to note that digital healthcare is not an end in itself - it is a means to offer healthcare differently. This can have various benefits, such as reducing travel time, easing the workload of healthcare providers, or improving the quality of care. However, it can also have drawbacks, as not everyone is equally adept at using a computer or smartphone, and it may lead to less personal contact.

**Agreement on Recording**

I would like to record this conversation. Is that okay with you?

**Introduction of participant**

Now, I'm interested in learning more about you. Could you briefly tell me who you are, and could you share something about where you are in the disease process?

**Urgency of Care**

[Slide 2: Headlines]

I'd like to start with some general questions about the current state of healthcare. I will show you a slide now. As you can see, staff shortages, rising costs...

1. Do you recognize this news? Do you believe that something really needs to change in healthcare to continue providing good care to all Dutch citizens, or do you think it will be fine as it is?

As the slide already indicates, Dutch healthcare is under pressure. This means that healthcare costs are rising, and staff shortages are growing. As a result, there is a need to think about how we can organize healthcare more efficiently while maintaining the quality of care.

The way healthcare is delivered needs to change. For example, by implementing digital healthcare. It also requires something from patients; they are expected to take more control of their own care, for instance, by being more self-sufficient in their recovery during the aftercare phase following treatment.

1. What do you think of the idea that patients should have more control or should take more control over a part of their disease process?
   *Prompt: What do you observe in your own environment?*

Now, I would like to talk about your situation as a patient. From previous research, we know that many people with cancer have a strong need for more support after treatment, especially during aftercare, and that this can be effectively provided through online aftercare. In this conversation, we would like to discuss with you how you view the use of online aftercare and what could either help or hinder you from using these programs.

[Slide 3: Case]

**Case**

To give you an idea of what online aftercare programs can entail, we'll provide an example of such a program:

Through an online aftercare program, people who have completed their cancer treatment can fill out a brief questionnaire on a website to assess how they are doing. This way, it can be determined what is going well and where they still need help or are experiencing difficulties. For example, based on the questionnaire, it might be identified that someone is still suffering from fatigue. The program covers topics such as returning to work, relationships with others (including sexuality and intimacy), anxiety and depressive feelings, dealing with pain, exercise, nutrition, and smoking cessation.

Based on the questionnaire, the program provides advice on which modules would be most valuable for a participant. If someone is experiencing fatigue, they can follow the fatigue module. This module includes:

• Information on how to deal with fatigue, sleep-wake rhythms, and thoughts about fatigue.
• Videos featuring the experiences of people with cancer on this topic, as well as advice from a clinical psychologist.
• Participants also receive assignments, such as daily tracking of an activity diary to gain insight into which activities are tiring or relaxing.

Now, I would like to discuss online aftercare in detail with you, how you feel about it, and what could help or hinder you in using it. You can keep the example program in mind.

**Goal Behavior: Use of Online Aftercare**

First, I am curious about:

1. You indicated when you signed up for this conversation that you have a need, or had a need, for support in your recovery and well-being during the aftercare phase. Can you specify in what areas that need lies?
2. To get a general impression of your experience, I would like to know to what extent you are already using online aftercare programs.
   <*If someone already has experience with this>*• What do you like about these programs?
   • What do you miss or what would you like to see differently?

**B. Psychological Capability - Knowledge, Memory, Attention, Decision Processes, Behavior Regulation**

Now, I would like to talk to you about how familiar you are with specific online aftercare programs.

1. Can you tell me to what extent you were already familiar with online aftercare programs before this conversation? What do you know about them?
2. What (digital) options do you know to actively work on your well-being and recovery?
3. To what extent do you know where to find these programs? If not, how would you like to be informed about these opportunities?
   *Prompt: e.g., through an oncologist, a general practitioner, social media, etc.
   Prompt: You mentioned earlier that you have already used X, how did you come across it?*
4. Have others pointed out the possibilities of digital support in your recovery during aftercare?
   *Prompt: Did your healthcare providers discuss it?
   Prompt: Or fellow patients?*

*<If the answer to question 4 is "no">*

1. From whom would you like to receive this information, so you would actually use online aftercare? And at what point would you like to receive this information?
   *Prompt: Timing; when in the process would you like to receive this information?*

**A. Reflective Motivation - Beliefs about Capabilities and Consequences, Roles, Identity, Intentions, Goals, Optimism**

I would now like to talk to you about your willingness and motivation to use online aftercare for your well-being and recovery.

1. You mentioned earlier that you mainly need [answer to question 1] in terms of aftercare. Do you think an online aftercare program can help you with this, or that you need it? If so, in what ways can it help you?
   *Prompt: Do you feel like you should use it?
   Prompt: Does it contribute to your recovery and well-being?
   Prompt: Do you think an online aftercare program suits you?
   Prompt: Would you be open to using online aftercare?*
2. Is using online aftercare programs something normal or common for you? Why or why not?
3. What benefits do you see in using online aftercare programs?
   *Prompt: What would happen if you did not use (online) aftercare programs?*
4. What disadvantages do you see in using online aftercare programs? Do the benefits outweigh the disadvantages for you?
   *Prompt: disadvantages compared to offline aftercare programs*
5. How do you think you would feel when using online aftercare programs?
   *Prompt: For example, feeling abandoned or empowered
   Prompt: what emotions might they trigger?*

**C. Physical Competence - Skills, Abilities, or Capabilities Acquired through Practice**

Not everyone can easily use online aftercare programs. Certain skills and abilities are required, and you must also be physically capable.

1. How easy or difficult do you think it would be for you to use online aftercare programs?
   *Prompt: Digital skills (how easy do you find it to log in to websites, such as online banking)?
   Prompt: What would help you in this regard?*
2. Do you need assistance with this?
   *Prompt: For example, from family or friends, an IT helpdesk, or an introductory course?*
3. Do you believe you are physically capable of using online aftercare?
   *Prompt: For example, do you have enough energy, endurance, and concentration during your treatment recovery to use it?*
4. What would help you in this regard?

**D. Physical Possibilities - Environmental Context and Resources**

In addition to skills, the use of online aftercare programs also requires certain things from patients. You need to engage with it on your own time and require specific resources, such as a computer and reliable Wi-Fi. I would like to discuss this with you now.

1. Do you have the time to use online aftercare programs?
2. Are you willing to pay for it yourself, or do you think an online aftercare program should be (partially) financed for you?
3. Do you have the necessary equipment and facilities to use online aftercare programs?
   *Prompt: ICT; stable Wi-Fi*
4. Are there other factors in your environment that could help or hinder you from using online aftercare programs?
   *Prompt: For example, automated reminders*
5. Do you have confidence that something can be done to address your obstacles?

**E. Social Opportunities - Social Influences such as Social Norms, Pressure, Conformity, Comparisons**

Now, I would like to continue talking with you about how your environment views the use of online aftercare. By your environment, I mean both your family and friends and the healthcare providers you have (or have had) for your treatment.

1. How does your environment view the use of online aftercare programs (as you estimate)?
   *Prompt: e.g., healthcare providers, family/friends*

To what extent does this influence your decision to use or not use online aftercare programs?

1. How does your environment view your recovery and well-being?
   *Prompt: Do you discuss this? Do they support you in this?*
2. To what extent do you think your environment would help/support you in applying the lessons learned from an online aftercare program?
   *Prompt: Can you discuss it with them? Would they remind you?*
3. Do you know other people who use online aftercare programs?

**F. Automatic Motivation - Emotions, Reinforcements such as Rewards, Punishments, Incentives**

Finally, I would like to discuss how an online aftercare program would need to be designed for you to use it effectively.

1. What aspects of the design of an online aftercare program would help you to use it?
   *Prompt: For example, in terms of design, certain rewards for using it, or ways to remind you to use it?*
2. Do you think you could make a habit of using online aftercare programs? What could help you with this?
   *Prompt: To what extent do you think you would remember to use online aftercare programs in your daily life?*
3. What do you need to integrate it into your daily life?

**Priorities**

I would like to conclude the conversation now. Finally, looking back on this conversation, if you had to identify the 2-3 most important things for you to use online aftercare, what would those be?
*Prompt: Of course, you've mentioned many things, but what are the top 2 most important things?*

**Conclusion**

Thank you very much for the conversation. We will process and analyze the conversations anonymously. The results will be published in a scientific article. We will send you the gift voucher via email soon.

If you have any questions or additional content to add later, please feel free to contact us. You have my email address.
