# Supplementary material for: What is needed for improved uptake and adoption of digital aftercare programs by cancer survivors: a mixed methods study applying the COM-B model
Source: J Cancer Surviv. 2024 Jul 4;20(1):323–35. doi: 10.1007/s11764-024-01635-x (PMC12906513; doi:10.1007/s11764-024-01635-x)
Supplement: Supplementary file 3 — Supplementary file3 (DOCX 44 KB) [file 11764_2024_1635_MOESM3_ESM.docx]

Supplementary File 3. Questionnaire

| **Question to respondents** | **Answer categories** | **Routing and recoding of answers** |
| --- | --- | --- |
| Background questions | | |
| 1. What is your gender? | 1. Male 2. Female 3. Non-binary 4. Prefer not to say |  |
| 1. What is your age (in years)? | Open question |  |
| 1. What is your marital status? | 1. Single (never married or in a registered partnership before) 2. In a relationship (not married and no registered partnership) 3. Married or in a registered partnership 4. Divorced 5. Widow or widower 6. Other, namely |  |
| 1. What is your highest completed education? | 1. Lower secondary vocational education or special secondary education (in Dutch: Lbo, vso, lts, leao, vbo, huishoudschool, ambachtsschool) 2. Preparatory vocational secondary education or remedial education (in Dutch: Vmbo, lwoo) 3. Middle general secondary education (in Dutch: Mavo, ulo, mulo) 4. Senior general secondary education (in Dutch: Havo, mms) 5. Pre-university education, higher civic school or lyceum (in Dutch: VWO, gymnasium, atheneym, hbs, lyceum) 6. Post-secondary vocational education (in Dutch: Mbo, mts, meao, middenstandsdiploma, pdb, mba) 7. Higher professional education (in Dutch: Hbo, hts, heao, kweekschool, associate degree) 8. Academic education, including postgraduate programs and doctoral research (in Dutch: WO) | - Secondary (vocational) education if answer category 1, 2, 3, 4 or 5 was checked - Post-secondary vocational education if answer category 6 was checked - Higher professional education or academic education if answer category 7 or 8 was checked |
| 1. Have you had difficulty making ends meet from your household income in the past 12 months? | 1. No, no difficulty at all 2. No, no difficulty, but I do need to watch my spending 3. Yes, some difficulty 4. Yes, significant difficulty 5. I don't know 6. Prefer not to say |  |
| 1. What type of cancer have you (had)? | 1. Pancreatic cancer 2. Bladder cancer 3. Breast cancer 4. Colon cancer 5. Skin cancer 6. Lung cancer 7. Lymph node cancer 8. Kidney cancer 9. Prostate cancer 10. Esophageal cancer 11. Other, namely |  |
| 1. In which stage of the illness are you? | 1. Undergoing treatment 2. Under control after treatment 3. Chronic phase 4. Cured 5. Palliative phase 6. I don't know 7. Other, namely |  |
| 1. How many years ago did you complete the treatment? Follow-up appointments are not included in the treatment period | 1. I am currently undergoing treatment 2. Less than one year ago 3. One to two years ago 4. Three to four years ago 5. Five to six years ago 6. Seven to eight years ago 7. More than eight years ago 8. I don't know |  |
| Challenges and complaints | | |
| 1. What complaints or challenges are you experiencing as a result of cancer or cancer treatment? (Multiple answers possible) | 1. Fatigue 2. Fear of cancer recurrence 3. Mood issues (such as sadness) 4. Dealing with pain (including neuropathy) 5. Coping with the illness and its processing 6. Problems with concentration 7. Relationships with others (including sexuality and intimacy) 8. (Returning to) work 9. Nutrition 10. Physical activity 11. Alcohol consumption 12. Quitting smoking 13. Desire for peer support |  |
| 1. To what extent do you agree with the following statement: "I think it is important to address my complaints or challenges to alleviate the resulting stress." If this varies by complaint or challenge, consider the one that is most important to you | 1. Completely agree 2. Agree 3. Neither agree nor disagree 4. Disagree 5. Completely disagree 6. I don't know |  |
| 1. What activities have you done to help with the complaints or challenges you are experiencing? (multiple answers possible) | 1. Visited a general practitioner (GP) 2. Visited a physiotherapist 3. Visited a psychologist 4. Visited a dietitian 5. Explored alternative therapies (e.g. acupuncture or homeopathy) 6. Attended a physical meeting of a patient association or foundation 7. Engaged in physical peer support (e.g. attended a gathering at a support center) 8. Searched for online information 9. Engaged in digital peer support (e.g. via Kanker.nl) 10. Visited social media platforms like Facebook 11. I have not taken any actions yet for the complaints and challenges I am experiencing |  |
| 1. For which complaints or challenges would you like (more) support? (multiple answers possible) | 1. Fatigue 2. Fear of cancer recurrence 3. Mood issues (such as sadness) 4. Dealing with pain (including neuropathy) 5. Coping with the illness and its processing 6. Problems with concentration 7. Relationships with others (including sexuality and intimacy) 8. (Returning to) work 9. Nutrition 10. Physical activity 11. Alcohol consumption 12. Quitting smoking 13. Desire for peer support 14. I don’t need any support for my complaints or challenges | - The questionnaire ended when answer category 14 was selected. The participants who selected this answer category were excluded from the analysis. |
| Familiarity with digital aftercare programs | | |
| 1. Were you already familiar with digital aftercare programs as explained in the video before starting this questionnaire? | 1. Yes 2. No | - If answer category 1 was selected, question 14 – 16 were shown - If answer category 2 was selected, questions 14 – 16 were skipped |
| 1. How did you learn about digital aftercare programs? (Multiple answers possible) | 1. Through the general practitioner (GP) 2. Through the medical specialist 3. Through the (oncology) nurse 4. Through another healthcare provider 5. Through a patient association or foundation 6. Through Kanker.nl 7. Through social media 8. Through Google or another search engine 9. Through friends / family 10. Through peers 11. Other, namely |  |
| 1. Have you ever used a digital aftercare program yourself? If yes, for which complaints or challenges? (Multiple answers possible) | 1. No, I have not (yet) used any digital aftercare programs 2. Yes: fatigue 3. Yes: fear of cancer recurrence 4. Yes: mood issues (such as sadness) 5. Yes: dealing with pain (including neuropathy) 6. Yes: coping with the illness and its processing 7. Yes: problems with concentration 8. Yes: relationships with others (including sexuality and intimacy) 9. Yes: (returning to) work 10. Yes: nutrition 11. Yes: physical activity 12. Yes: alcohol consumption 13. Yes: quitting smoking 14. Yes: desire for peer support 15. Yes: for another complaint or challenge, namely |  |
| 1. To what extent do you agree with the following statement: "The digital aftercare programs I have used are beneficial for addressing my complaints or challenges" | 1. Completely agree 2. Agree 3. Neither agree nor disagree 4. Disagree 5. Completely disagree 6. I don't know |  |
| 1. To what extent do you agree with the following statement: "I would use digital aftercare programs for the complaints or challenges I am experiencing due to cancer or cancer treatment" | 1. Completely agree 2. Agree 3. Neither agree nor disagree 4. Disagree 5. Completely disagree 6. I don't know |  |
| 1. How would you prefer to hear about digital aftercare programs? (Multiple answers possible) | 1. Through the general practitioner (GP) 2. Through the medical specialist 3. Through the (oncology) nurse 4. Through the case manager 5. Through a patient association 6. Through Kanker.nl 7. Through social media such as private Facebook groups 8. Through search engines like Google 9. Through friends or family 10. I do not want to hear about digital aftercare programs 11. I don't know 12. In another way, namely | - If answer category 10 was selected, question 19 was not shown. |
| 1. At what point would you have preferred to hear about the existence of digital aftercare programs? (Multiple answers possible) | 1. During the diagnosis phase 2. During the treatment 3. Immediately after completing the treatment 4. A few weeks after completing the treatment 5. I don't know 6. At another time, namely |  |
| Attitude towards digital aftercare programs | | |
| 1. To what extent do you agree with the following statement: "Digital aftercare programs can help me with the complaints or challenges I am experiencing due to cancer or cancer treatment" | 1. Completely agree 2. Agree 3. Neither agree nor disagree 4. Disagree 5. Completely disagree 6. I don't know |  |
| 1. For which complaints or challenges would you find it useful to use a digital aftercare program? (Multiple answers possible) | 1. Fatigue 2. Fear of cancer recurrence 3. Mood issues (such as sadness) 4. Dealing with pain (including neuropathy) 5. Coping with the illness and its processing 6. Problems with concentration 7. Relationships with others (including sexuality and intimacy) 8. (Returning to) work 9. Nutrition 10. Physical activity 11. Alcohol consumption 12. Quitting smoking 13. Desire for peer support 14. I don't know 15. I do not need help or support through a digital aftercare program 16. Another complaint or challenge, namely |  |
| 1. Which components of a digital aftercare program would be useful for you? (Multiple answers possible) | 1. Information, tips, and advice 2. Experiences of other people who have had cancer 3. Contact with other people who have had cancer 4. Assignments to work on individually 5. Referrals to more information or help 6. I don't know 7. No components 8. Another component, namely |  |
| 1. What do you see as the main benefits of using digital aftercare programs compared to physical support? (You can select up to five answers) | 1. Being in control of when you use it 2. Being able to pause in between 3. Being able to review information 4. Not having to go to a healthcare provider 5. Saves travel time 6. Saves costs for me as a patient 7. Saves costs for healthcare 8. Provides support in the post-treatment phase 9. Being able to work independently 10. Being able to start immediately (without waiting list) 11. Being able to work anonymously (e.g. with potentially sensitive topics like sexuality) 12. I do not see any benefits 13. I don't know 14. Another benefit, namely |  |
| 1. What do you see as the main disadvantages of using digital aftercare compared to physical support? (You can select up to three answers) | 1. No possibility of personal contact 2. No possibility of asking questions 3. It takes strength to persist in using the program 4. Need to be tech-savvy 5. Having to work independently on complaints or challenges 6. A healthcare provider could better assist me with my complaints or challenges 7. I do not see any disadvantages 8. I don't know 9. Another disadvantage, namely |  |
| 1. Do you think the benefits of using digital aftercare programs would outweigh the disadvantages for you? | 1. Yes 2. No 3. I don’t know |  |
| 1. To what extent do you agree with the following statement: "I would like to address my complaints or challenges independently and online, without the involvement of a healthcare provider or someone else" | 1. Completely agree 2. Agree 3. Neither agree nor disagree 4. Disagree 5. Completely disagree 6. I don't know |  |
| 1. To what extent do agree with the following statement: "Using digital aftercare programs is a good fit for me as an individual" | 1. Completely agree 2. Agree 3. Neither agree nor disagree 4. Disagree 5. Completely disagree 6. I don't know |  |
| 1. To what extent do you agree with the following statement: "Using digital aftercare programs would be something normal for me" | 1. Completely agree 2. Agree 3. Neither agree nor disagree 4. Disagree 5. Completely disagree 6. I don't know |  |
| **Capability of using digital aftercare programs** | | |
| 1. To what extent do you agree with the following statement: "I believe I have sufficient digital skills to use digital aftercare programs" | 1. Completely agree 2. Agree 3. Neither agree nor disagree 4. Disagree 5. Completely disagree 6. I don't know |  |
| 1. Would you like assistance with using digital aftercare programs? If yes, what kind of assistance? (Multiple answers possible) | 1. No, I do not want any assistance 2. Yes, a digital helpdesk via email or chat 3. Yes, a physical helpdesk at the hospital or with a healthcare provider (e.g. a general practitioner or physiotherapist) 4. Yes, a phone number that I can call 5. Yes, a course on using digital aftercare programs 6. Yes, assistance from people in my surroundings (e.g. family, friends, or colleagues) 7. Yes, assistance via the library 8. Yes, an explanation within the digital aftercare program 9. I don't know 10. Yes, another form of assistance, namely |  |
| 1. What factors could prevent you from using digital aftercare programs? (Multiple answers possible) | 1. I don't have a good internet connection 2. I don't have a computer, smartphone, or tablet 3. I have little money to purchase a digital aftercare program 4. I have little time 5. I have little energy 6. I have difficulty concentrating 7. Concerns about privacy 8. Doubts about the program's reliability (e.g. information and advice) 9. Doubts about the program's effectiveness 10. Doubts about whether the program aligns with the advice of my healthcare providers 11. There are no factors that would prevent me from using digital aftercare programs 12. Other, namely |  |
| 1. How much would you be willing to pay for the use of a digital aftercare program (in euros)? Please enter '0' if you do not want to pay for the use of digital aftercare programs. | Open question |  |
| 1. Would it be a requirement for you to have digital aftercare programs fully covered by your health insurance in order for you to use these programs? | 1. Yes 2. No 3. I don’t know |  |
| Social environment and digital aftercare programs | | |
| 1. Do you know other people who use digital aftercare programs? | 1. Yes 2. No 3. I don’t know |  |
| 1. How do you expect your healthcare providers to view the use of digital aftercare programs? | 1. Very positively 2. Positively 3. Neutral 4. Negatively 5. Very negatively 6. I don't know |  |
| 1. To what extent do you agree with the following statement: "The opinion of my healthcare providers about digital aftercare programs would influence my decision to use digital aftercare programs" | 1. Completely agree 2. Agree 3. Neither agree nor disagree 4. Disagree 5. Completely disagree 6. I don't know |  |
| 1. How do you expect the people in your surroundings (e.g., friends, family, or colleagues) to view the use of digital aftercare programs? | 1. Very positively 2. Positively 3. Neutral 4. Negatively 5. Very negatively 6. I don't know |  |
| 1. To what extent do you agree with the following statement: "The opinion of the people in my surroundings about digital aftercare programs would influence my decision to use digital aftercare programs" | 1. Completely agree 2. Agree 3. Neither agree nor disagree 4. Disagree 5. Completely disagree 6. I don't know |  |
| 1. To what extent do you concur with the following statement: "I feel the need for support from people around me when applying the insights gained from a digital aftercare program"? | 1. Completely agree 2. Agree 3. Neither agree nor disagree 4. Disagree 5. Completely disagree 6. I don't know |  |
| 1. To what extent do you agree with the following statement: "I expect that the people in my environment can support me in using the things I learn in a digital aftercare program"? | 1. Completely agree 2. Agree 3. Neither agree nor disagree 4. Disagree 5. Completely disagree 6. I don't know |  |
| Daily use of digital aftercare programs | | |
| 1. How easy or difficult would it be for you to regularly use a digital aftercare program and stick to it (e.g., a few times per week)? | 1. Very easy 2. Easy 3. Neither easy nor difficult 4. Difficult 5. Very difficult 6. I don't know |  |
| 1. What would help you to regularly use and maintain a digital aftercare program (e.g., a few times per week)? (Multiple answers possible) | 1. Regular reminders 2. Insight into the duration of the program and which part I have already completed 3. The program provides feedback on my activities (e.g., compliments or tips) 4. The program is tailored to my personal situation (e.g., type of cancer) 5. Ability to set clear goals in the program 6. The program is accessible on both a computer and a tablet or phone 7. Rewards (e.g., earning points) 8. Digital contact with a healthcare provider or coach 9. Digital contact with someone who has also had cancer 10. I don't know 11. Other, namely |  |
| 1. What is your view of digital aftercare programs as explained in the video? | 1. Very positive 2. Positive 3. Neutral 4. Negative 5. Very negative |  |
