# Supplementary material for: What is needed for improved uptake and adoption of digital aftercare programs by cancer survivors: a mixed methods study applying the COM-B model
Source: J Cancer Surviv. 2024 Jul 4;20(1):323–35. doi: 10.1007/s11764-024-01635-x (PMC12906513; doi:10.1007/s11764-024-01635-x)
Supplement: Supplementary file 5 — Supplementary file5 (DOCX 78 KB) [file 11764_2024_1635_MOESM5_ESM.docx]

Supplementary File 5. Descriptive analyses

Table 1. Participants’ characteristics

|  | **Total** | |
| --- | --- | --- |
|  |  | |
| **Study population frequencies (n)** | **N = 213** | |
|  | ***n*** | **%** |
| Gender | | |
| *Male* | 70 | 32.9 |
| *Female* | 141 | 66.2 |
| *Non-binary* | 2 | 0.9 |
| Age | | |
| *Mean (SD, min-max)* | 71 (10.6, 23 – 94) | |
| Marital status | | |
| *Single* | 29 | 13.6 |
| *Married or registered partnership* | 123 | 57.7 |
| *Divorced* | 15 | 7.0 |
| *In a relationship (not married or in a registered partnership)* | 31 | 14.6 |
| *Widow(er)* | 14 | 6.6 |
| *Other* | 1 | 0.5 |
| Educational level | | |
| *Secondary (vocational) education* | 43 | 20.2 |
| *Post-secondary vocational education* | 52 | 24.4 |
| *Higher professional education or academic education* | 118 | 55.4 |
| Difficulty making ends meet from household income in the past 12 months | | |
| *Yes, significant difficulty* | 10 | 4.7 |
| *Yes, some difficulty* | 38 | 17.8 |
| *No, no difficulty, but I need to watch my expenses* | 78 | 36.6 |
| *No, no difficulty at all* | 86 | 40.4 |
| *I would rather not say* | 1 | 0.5 |
| Cancer type | | |
| *Breast cancer* | 51 | 23.9 |
| *Colorectal cancer* | 22 | 10.3 |
| *Bladder cancer* | 20 | 9.4 |
| *Prostate cancer* | 16 | 7.5 |
| *Throat or laryngeal cancer* | 13 | 6.1 |
| *Esophageal cancer* | 11 | 5.2 |
| *Ovarian cancer* | 8 | 3.8 |
| *Skin cancer* | 8 | 3.8 |
| *Multiple types of cancer* | 8 | 3.8 |
| *Uterine or cervical cancer* | 7 | 3.3 |
| *Lymph node cancer* | 7 | 3.3 |
| *Lung cancer* | 6 | 2.8 |
| *Brain tumor* | 5 | 2.3 |
| *Pancreatic cancer* | 3 | 1.4 |
| *Oral cancer* | 3 | 1.4 |
| *Bone marrow cancer* | 2 | 0.9 |
| *Head and neck cancer* | 2 | 0.9 |
| *Nasal or sinus cancer* | 2 | 0.9 |
| *Kidney cancer* | 2 | 0.9 |
| *Sarcoma* | 2 | 0.9 |
| *Thyroid cancer* | 2 | 0.9 |
| *Vulvar cancer* | 2 | 0.9 |
| *Acute leukemia* | 1 | 0.5 |
| *Stomach cancer* | 1 | 0.5 |
| *Neuroendocrine carcinoma* | 1 | 0.5 |
| *Salivary gland cancer* | 1 | 0.5 |
| *Pseudomyxoma Peritonei* | 1 | 0.5 |
| Stage of the illness | | |
| *Undergoing treatment* | 28 | 13.1 |
| *Under control after treatment* | 128 | 60.1 |
| *Chronic phase* | 8 | 3.8 |
| *Cured* | 29 | 13.6 |
| *Palliative phase* | 14 | 6.6 |
| *I don't know* | 1 | 0.5 |
| *Other* | 5 | 2.3 |
| Time since treatment completion | | |
| *Currently undergoing treatment* | 34 | 16.0 |
| *Less than one year ago* | 46 | 21.6 |
| *One to two years ago* | 53 | 24.9 |
| *Three to four years ago* | 37 | 17.4 |
| *Five to six years ago* | 14 | 6.6 |
| *Seven to eight years ago* | 7 | 3.3 |
| *More than eight years ago* | 22 | 10.3 |

Table 2. Challenges and complaints

|  | **Total** | |
| --- | --- | --- |
|  |  | |
| **Study population frequencies (n)** | **N = 213** | |
|  | ***n*** | **%** |
| What complaints or challenges are you experiencing as a result of cancer or cancer treatment? (Multiple answers possible) | | |
| *Fatigue* | 162 | 76.1 |
| *Fear of cancer recurrence* | 119 | 55.9 |
| *Problems with concentration* | 102 | 47.9 |
| *Dealing with pain (including neuropathy)* | 100 | 46.9 |
| *Relationships with others (including sexuality and intimacy)* | 88 | 41.3 |
| *Coping with the illness and its processing* | 82 | 38.5 |
| *Mood issues (such as sadness)* | 77 | 36.2 |
| *Physical activity* | 77 | 36.2 |
| *(Returning to) work* | 68 | 31.9 |
| *Nutrition* | 50 | 23.5 |
| *Desire for peer support* | 40 | 18.8 |
| *Quitting smoking* | 10 | 4.7 |
| *Alcohol consumption* | 8 | 3.8 |
| To what extent do you agree with the following statement: "I think it is important to address my complaints or challenges to alleviate the resulting stress” | | |
| *Completely agree* | 124 | 58.2 |
| *Agree* | 72 | 33.8 |
| *Neither agree nor disagree* | 16 | 7.5 |
| *Disagree* | 0 | 0.0 |
| *Completely disagree* | 0 | 0.0 |
| *I do not know* | 1 | 0.5 |
| What activities have you done to help with the complaints or challenges you are experiencing? (multiple answers possible) | | |
| *Searched for online information* | 143 | 67.1 |
| *Visited a physiotherapist* | 130 | 61.0 |
| *Visited a general practitioner (GP)* | 108 | 50.7 |
| *Engaged in digital peer support (e.g., via Kanker.nl)* | 96 | 45.1 |
| *Visited a psychologist* | 91 | 42.7 |
| *Visited social media platforms like Facebook* | 91 | 42.7 |
| *Visited a dietitian* | 51 | 23.9 |
| *Explored alternative therapies (e.g., acupuncture or homeopathy)* | 49 | 23.0 |
| *Engaged in physical peer support (e.g., attended a gathering at a support center)* | 45 | 21.1 |
| *Attended a physical meeting of a patient association or foundation* | 44 | 20.7 |
| *I have not taken any actions yet for the complaints and challenges I am experiencing* | 7 | 3.3 |
| For which complaints or challenges would you like (more) support? (multiple answers possible) | | |
| *Fatigue* | 105 | 49.3 |
| *Fear of cancer recurrence* | 83 | 39.0 |
| *Coping with the illness and its processing* | 63 | 29.6 |
| *Problems with concentration* | 58 | 27.2 |
| *Dealing with pain (including neuropathy)* | 56 | 26.3 |
| *Physical activity* | 56 | 26.3 |
| *Relationships with others (including sexuality and intimacy)* | 50 | 23.5 |
| *Mood issues (such as sadness)* | 45 | 21.1 |
| *Nutrition* | 42 | 19.7 |
| *(Returning to) work* | 37 | 17.4 |
| *Desire for peer support* | 21 | 9.9 |
| *Quitting smoking* | 6 | 2.8 |
| *Alcohol consumption* | 5 | 2.3 |

Table 3. Familiarity with digital aftercare programs

|  | **Total** | |
| --- | --- | --- |
|  |  | |
| **Study population frequencies (n)** | **N = 213** | |
|  | ***n*** | **%** |
| Were you already familiar with digital aftercare programs as explained in the video before starting this questionnaire? | | |
| *Yes* | 33 | 15.5 |
| *No* | 180 | 84.5 |
| How did you learn about digital aftercare programs? (Multiple answers possible) (N = 33) | | |
| *Through Kanker.nl* | 11 | 33.3 |
| *Through the (oncology) nurse* | 8 | 24.2 |
| *Through the medical specialist* | 7 | 21.2 |
| *Through social media* | 6 | 18.2 |
| *Through another healthcare provider* | 5 | 15.2 |
| *Through a patient association or foundation* | 4 | 12.2 |
| *Through peers* | 3 | 9.1 |
| *Through Google or another search engine* | 2 | 6.1 |
| *Through friends / family* | 2 | 6.1 |
| *Through the general practitioner* | 0 | 0.0 |
| Have you ever used a digital aftercare program yourself? If yes, for which complaints or challenges? (Multiple answers possible) (N = 33) | | |
| *No, I have not (yet) used any digital aftercare programs* | 12 | 36.4 |
| *Yes: fatigue* | 16 | 48.5 |
| *Yes: fear of cancer recurrence* | 5 | 15.2 |
| *Yes: coping with the illness and its processing* | 5 | 15.2 |
| *Yes: problems with concentration* | 4 | 12.1 |
| *Yes: dealing with pain (including neuropathy)* | 2 | 6.1 |
| *Yes: relationships with others (including sexuality and intimacy)* | 2 | 6.1 |
| *Yes: (returning to) work* | 1 | 3.0 |
| *Yes: physical activity* | 1 | 3.0 |
| *Yes: desire for peer support* | 1 | 3.0 |
| *Yes: mood issues (such as sadness)* | 0 | 0.0 |
| *Yes: nutrition* | 0 | 0.0 |
| *Yes: alcohol consumption* | 0 | 0.0 |
| *Yes: quitting smoking* | 0 | 0.0 |
| To what extent do you agree with the following statement: "The digital aftercare programs I have used are beneficial for addressing my complaints or challenges" (N = 21) | | |
| *Completely agree* | 2 | 9.5 |
| *Agree* | 8 | 38.1 |
| *Neither agree nor disagree* | 8 | 38.1 |
| *Disagree* | 2 | 9.5 |
| *Completely disagree* | 1 | 4.8 |
| *I do not know* | 0 | 0.0 |
| To what extent do you agree with the following statement: "I would use digital aftercare programs for the complaints or challenges I am experiencing due to cancer or cancer treatment" (N = 143) | | |
| *Completely agree* | 33 | 23.1 |
| *Agree* | 59 | 41.3 |
| *Neither agree nor disagree* | 31 | 21.7 |
| *Disagree* | 6 | 4.2 |
| *Completely disagree* | 5 | 3.5 |
| *I do not know* | 9 | 6.3 |
| How would you prefer to hear about digital aftercare programs? (Multiple answers possible) | | |
| *Through the general practitioner (GP)* | 99 | 46.5 |
| *Through the medical specialist* | 131 | 61.5 |
| *Through the (oncology) nurse* | 127 | 59.6 |
| *Through Kanker.nl* | 84 | 39.4 |
| *Through a patient association* | 52 | 24.4 |
| *Through the case manager* | 41 | 19.2 |
| *Through social media such as private Facebook groups* | 40 | 18.8 |
| *Through search engines like Google* | 29 | 13.6 |
| *Through friends or family* | 5 | 2.3 |
| *I do not know* | 6 | 2.8 |
| *I do not want to hear about digital aftercare programs* | 2 | 0.9 |
| At what point would you have preferred to hear about the existence of digital aftercare programs? (Multiple answers possible) | | |
| Immediately after completing the treatment | 115 | 54.0 |
| During the treatment | 93 | 43.7 |
| A few weeks after completing the treatment | 60 | 28.2 |
| During the diagnosis phase | 34 | 16.0 |
| I do not know | 13 | 6.1 |

Table 4. Attitude towards digital aftercare programs

|  | **Total** | |
| --- | --- | --- |
|  |  | |
| **Study population frequencies (n)** | **N = 213** | |
|  | ***n*** | **%** |
| To what extent do you agree with the following statement: "Digital aftercare programs can help me with the complaints or challenges I am experiencing due to cancer or cancer treatment" | | |
| Completely agree | 48 | 22.5 |
| Agree | 82 | 38.5 |
| Neither agree nor disagree | 50 | 23.5 |
| Disagree | 4 | 1.9 |
| Completely disagree | 3 | 1.4 |
| I do not know | 26 | 12.2 |
| For which complaints or challenges would you find it useful to use a digital aftercare program? (Multiple answers possible) | | |
| Fatigue | 132 | 62.0 |
| Fear of cancer recurrence | 102 | 47.9 |
| Coping with the illness and its processing | 90 | 42.3 |
| Dealing with pain (including neuropathy) | 84 | 39.4 |
| Problems with concentration | 72 | 33.8 |
| Nutrition | 70 | 32.9 |
| Relationships with others (including sexuality and intimacy) | 68 | 31.9 |
| Mood issues (such as sadness) | 66 | 31.0 |
| Physical activity | 66 | 31.0 |
| Desire for peer support | 42 | 19.7 |
| (Returning to) work | 41 | 19.2 |
| Alcohol consumption | 12 | 5.6 |
| Quitting smoking | 11 | 5.2 |
| I do not need help or support through a digital aftercare program | 9 | 4.2 |
| I do not know | 7 | 3.3 |
| Which components of a digital aftercare program would be useful for you? (Multiple answers possible) | | |
| Information, tips, and advice | 172 | 80.8 |
| Experiences of other people who have had cancer | 126 | 59.2 |
| Referrals to more information or help | 113 | 53.1 |
| Assignments to work on individually | 99 | 46.5 |
| Contact with other people who have had cancer | 74 | 34.7 |
| I do not know | 10 | 4.7 |
| No components | 6 | 2.8 |
| What do you see as the main advantages of using digital aftercare compared to physical support? (You can select up to five answers) | | |
| Being in control of when you use it | 170 | 79.8 |
| Being able to review information | 132 | 62.0 |
| Being able to start immediately (without waiting list) | 80 | 37.6 |
| Being able to work independently | 75 | 35.2 |
| Provides support in the post-treatment phase | 68 | 31.9 |
| Being able to pause in between | 66 | 31.0 |
| Not having to go to a healthcare provider | 40 | 18.8 |
| Being able to work anonymously (e.g., with potentially sensitive topics like sexuality) | 40 | 18.8 |
| Saves travel time | 30 | 14.1 |
| Saves costs for healthcare | 28 | 13.1 |
| Saves costs for me as a patient | 24 | 11.3 |
| I do not see any benefits | 8 | 3.8 |
| I do not know | 6 | 2.8 |
| What do you see as the main disadvantages of using digital aftercare compared to physical support? (You can select up to three answers) | | |
| No possibility of personal contact | 133 | 62.4 |
| No possibility of asking questions | 106 | 49.8 |
| It takes strength to persist in using the program | 76 | 35.7 |
| Having to work independently on complaints or challenges | 29 | 13.6 |
| Need to be tech-savvy | 24 | 11.3 |
| A healthcare provider could better assist me with my complaints or challenges | 20 | 9.4 |
| I do not see any disadvantages | 20 | 9.4 |
| I do not know | 10 | 4.7 |
| Do you think the benefits of using digital aftercare programs would outweigh the disadvantages for you? | | |
| Yes | 114 | 53.5 |
| No | 23 | 10.8 |
| I do not know | 76 | 35.7 |
| To what extent do you agree with the following statement: "I would like to address my complaints or challenges independently and online, without the involvement of a healthcare provider or someone else" | | |
| Completely agree | 35 | 16.4 |
| Agree | 87 | 40.8 |
| Neither agree nor disagree | 48 | 22.5 |
| Disagree | 19 | 8.9 |
| Completely disagree | 12 | 5.6 |
| I do not know | 12 | 5.6 |
| To what extent do you agree with the following statement: "Using digital aftercare programs is a good fit for me as an individual" | | |
| Completely agree | 26 | 12.2 |
| Agree | 86 | 40.4 |
| Neither agree nor disagree | 54 | 25.4 |
| Disagree | 20 | 9.4 |
| Completely disagree | 13 | 6.1 |
| I do not know | 14 | 6.6 |
| To what extent do you agree with the following statement: "Using digital aftercare programs would be something normal for me" | | |
| Completely agree | 28 | 13.1 |
| Agree | 82 | 38.5 |
| Neither agree nor disagree | 60 | 28.2 |
| Disagree | 12 | 5.6 |
| Completely disagree | 12 | 5.6 |
| I do not know | 19 | 8.9 |

Table 5. Capability of using digital aftercare programs

|  | **Total** | |
| --- | --- | --- |
|  |  | |
| **Study population frequencies (n)** | **N = 213** | |
|  | ***n*** | **%** |
| To what extent do you agree with the following statement: "I believe I have sufficient digital skills to use digital aftercare programs" | | |
| Completely agree | 118 | 55.4 |
| Agree | 71 | 33.3 |
| Neither agree nor disagree | 11 | 5.2 |
| Disagree | 2 | 0.9 |
| Completely disagree | 5 | 2.3 |
| I do not know | 6 | 2.8 |
| Would you like assistance with using digital aftercare programs? If yes, what kind of assistance? (Multiple answers possible) | | |
| No, I do not want any assistance | 85 | 39.9 |
| Yes, a digital helpdesk via email or chat | 60 | 28.2 |
| Yes, a phone number that I can call | 35 | 16.4 |
| Yes, an explanation within the digital aftercare program | 30 | 14.1 |
| Yes, a physical helpdesk at the hospital or with a healthcare provider (e.g., a general practitioner or physiotherapist) | 21 | 9.9 |
| Yes, assistance from people in my surroundings (e.g., family, friends, or colleagues) | 7 | 3.3 |
| Yes, a course on using digital aftercare programs | 6 | 2.8 |
| Yes, assistance via the library | 2 | 0.9 |
| I do not know | 28 | 13.1 |
| What factors could prevent you from using digital aftercare programs? (Multiple answers possible) | | |
| Doubts about the program's effectiveness | 64 | 30.0 |
| I have little energy | 58 | 27.2 |
| I have difficulty concentrating | 48 | 22.5 |
| Concerns about privacy | 32 | 15.0 |
| Doubts about the program's reliability (e.g., information and advice) | 29 | 13.6 |
| I have little money to purchase a digital aftercare program | 26 | 12.2 |
| Doubts about whether the program aligns with the advice of my healthcare providers | 26 | 12.2 |
| I have little time | 9 | 4.2 |
| I don't have a good internet connection | 7 | 3.3 |
| I don't have a computer, smartphone, or tablet | 3 | 1.4 |
| There are no factors that would prevent me from using digital aftercare programs | 56 | 26.3 |
| How much would you be willing to pay for the use of a digital aftercare program (in euros)? Please enter '0' if you do not want to pay for the use of digital aftercare programs. | | |
| % that filled in 0 euro (and therefore did not want to pay for digital aftercare programs) | 166 | 77.9 |
| Mean (SD, min-max) (of those who were willing to pay for digital aftercare programs, *n*=47^1^) | 48.8 (36.6, 10 – 150) | |
| Would it be a requirement for you to have digital aftercare programs fully covered by your health insurance in order for you to use these programs? | | |
| Yes | 128 | 60.1 |
| No | 39 | 18.3 |
| I do not know | 46 | 21.6 |

^1^One participant was excluded from the analysis as an outlier due to a significantly higher response.

Table 6. Social environment and digital aftercare programs

|  | **Total** | |
| --- | --- | --- |
|  |  | |
| **Study population frequencies (n)** | **N =** | |
|  | ***n*** | **%** |
| Do you know other people who use digital aftercare programs? | | |
| Yes | 12 | 5.6 |
| No | 184 | 86.4 |
| I don’t know | 17 | 8.0 |
| How do you expect your healthcare providers to view the use of digital aftercare programs? | | |
| Very positively | 21 | 9.9 |
| Positively | 102 | 47.9 |
| Neutral | 43 | 20.2 |
| Negatively | 4 | 1.9 |
| Very negatively | 1 | 0.5 |
| I do not know | 42 | 19.7 |
| To what extent do you agree with the following statement: "The opinion of my healthcare providers about digital aftercare programs would influence my decision to use digital aftercare programs" | | |
| Completely agree | 11 | 5.2 |
| Agree | 69 | 32.4 |
| Neither agree nor disagree | 54 | 25.4 |
| Disagree | 42 | 19.7 |
| Completely disagree | 23 | 10.8 |
| I do not know | 14 | 6.6 |
| How do you expect the people in your surroundings (e.g., friends, family, or colleagues) to view the use of digital aftercare programs? | | |
| Very positively | 18 | 8.5 |
| Positively | 81 | 38.0 |
| Neutral | 68 | 31.9 |
| Negatively | 3 | 1.4 |
| Very negatively | 0 | 0.0 |
| I do not know | 43 | 20.2 |
| To what extent do you agree with the following statement: "The opinion of the people in my surroundings about digital aftercare programs would influence my decision to use digital aftercare programs" | | |
| Completely agree | 1 | 0.5 |
| Agree | 13 | 6.1 |
| Neither agree nor disagree | 46 | 21.6 |
| Disagree | 76 | 35.7 |
| Completely disagree | 68 | 31.9 |
| I do not know | 9 | 4.2 |
| To what extent do you concur with the following statement: "I feel the need for support from people around me when applying the insights gained from a digital aftercare program"? | | |
| Completely agree | 7 | 3.3 |
| Agree | 69 | 32.4 |
| Neither agree nor disagree | 57 | 26.8 |
| Disagree | 37 | 17.4 |
| Completely disagree | 17 | 8.0 |
| I do not know | 26 | 12.2 |
| To what extent do you agree with the following statement: "I expect that the people in my environment can support me in using the things I learn in a digital aftercare program"? | | |
| Completely agree | 7 | 3.3 |
| Agree | 74 | 34.7 |
| Neither agree nor disagree | 56 | 26.3 |
| Disagree | 30 | 14.1 |
| Completely disagree | 12 | 5.6 |
| I do not know | 34 | 16.0 |

Table 7. Daily use of digital aftercare programs

|  | **Total** | |
| --- | --- | --- |
|  |  | |
| **Study population frequencies (n)** | **N =** | |
|  | ***n*** | **%** |
| How easy or difficult would it be for you to regularly use a digital aftercare program and stick to it (e.g., a few times per week)? | | |
| Very easy | 16 | 7.5 |
| Easy | 54 | 25.4 |
| Neither easy nor difficult | 78 | 36.6 |
| Difficult | 38 | 17.8 |
| Very difficult | 6 | 2.8 |
| I do not know | 21 | 9.9 |
| What would help you to regularly use and maintain a digital aftercare program (e.g., a few times per week)? (Multiple answers possible) | | |
| The program is tailored to my personal situation (e.g., type of cancer) | 134 | 62.9 |
| Insight into the duration of the program and which part I have already completed | 124 | 58.2 |
| The program provides feedback on my activities (e.g., compliments or tips) | 91 | 42.7 |
| Digital contact with a healthcare provider or coach | 87 | 40.8 |
| The program is accessible on both a computer and a tablet or phone | 85 | 39.9 |
| Regular reminders | 73 | 34.3 |
| Ability to set clear goals in the program | 73 | 34.3 |
| Digital contact with someone who has also had cancer | 41 | 19.2 |
| Rewards (e.g., earning points) | 19 | 8.9 |
| I do not know | 24 | 11.3 |
| What is your view of digital aftercare programs as explained in the video? | | |
| Very positive | 28 | 13.1 |
| Positive | 105 | 49.3 |
| Neutral | 69 | 32.4 |
| Negative | 8 | 3.8 |
| Very negative | 3 | 1.4 |
