# Supplementary material for: What is needed for improved uptake and adoption of digital aftercare programs by cancer survivors: a mixed methods study applying the COM-B model
Source: J Cancer Surviv. 2024 Jul 4;20(1):323–35. doi: 10.1007/s11764-024-01635-x (PMC12906513; doi:10.1007/s11764-024-01635-x)
Supplement: Supplementary file 6 — Supplementary file6 (DOCX 42 KB) [file 11764_2024_1635_MOESM6_ESM.docx]

Supplementary File 6. Explorative analyses regarding possible influencing variables as conducted in SPSS

Table 1: Results of the binary regression analysis results for predicting answer to the question: “*Were you already familiar with digital aftercare programs as explained in the video before starting this questionnaire?*” (Yes or no)

| Predictor variable^1^ | Wald Chi-square statistic | *P*-value |
| --- | --- | --- |
| Marital status | 1.05 | .31 |
| Age | 3.80 | .05 |
| Income | 0.18 | .68 |
| Educational level | 4.49 | .11 |
| Type of cancer | 5.66 | .23 |
| Duration since treatment completion | 0.70 | .88 |

^1 For the explorative analyses, the variables ‘Marital status’; ‘Income’; and ‘Duration since treatment completion’ were recoded. Marital status was recoded into two categories: 1) With partner: married or registered partnership (n=123, 57.7%); in a relationship (not married or in a registered partnership (n=31, 14.6%) and 2) Without partner: single (n=29, 13.6%); divorced (n=15, 7.0%); widow(er) (n=14, 6.4%). Other (n=1, 0.5%); Income was recoded into two categories: 1) Yes: significant difficulty (n= 10, 4.7%); some difficulty (n=38, 17.8%) and 2) No: no difficulty, but I need to watch my expenses (n=78, 36.6%); no difficulty (n=86, 40.4%). I would rather not say (n=1, .5%); Duration since treatment completion was recoded into four categories: 1) Currently undergoing treatment (n=34, 16.0%); 2) Less than one year ago (n=46 , 21.6%); 3) One to two years ago (n=53 , 24.9%); 4) Three years or more ago (n=80, 37.6%). For the variable ‘Type of cancer’ the following categories were used: breast cancer, colon cancer, bladder cancer, prostate cancer, and esophageal cancer.^

Table 2: Results of the binary regression analysis results for predicting answers to the question: “*How would you prefer to hear about digital aftercare programs*?” (Multiple answers possible)

| Answer option | Predictor variable | Wald Chi-Square statistic | P-value | Adjusted p-value^1^ (where applicable) |
| --- | --- | --- | --- | --- |
| Through the general practitioner | Marital status | 0.18 | .67 |  |
|  | Age | 0.23 | .63 |  |
|  | Income | 0.02 | .90 |  |
|  | Educational level | 1.98 | .37 |  |
|  | Type of cancer | 3.88 | .42 |  |
|  | Duration since treatment completion | 5.57 | .13 |  |
| Through the medical specialist | Marital status | 0.25 | .62 |  |
|  | Age | 7.56 | .006** | .18 |
|  | Income | 1.32 | .25 |  |
|  | Educational level | 2.48 | .29 |  |
|  | Type of cancer | 15.55 | .004** | .18 |
|  | Duration since treatment completion | 1.28 | .74 |  |
| Through the (oncology) nurse | Marital status | 2.72 | .10 |  |
|  | Age | 3.53 | .06 |  |
|  | Income | 0.41 | .52 |  |
|  | Educational level | 10.36 | .006** | .18 |
|  | Type of cancer | 7.29 | .12 |  |
|  | Duration since treatment completion | 1.94 | .59 |  |
| Through the case manager | Marital status | 1.82 | .18 |  |
|  | Age | 0.13 | .72 |  |
|  | Income | 1.98 | .16 |  |
|  | Educational level | 0.48 | .79 |  |
|  | Type of cancer | 0.89 | .93 |  |
|  | Duration since treatment completion | 2.79 | .42 |  |
| Through a patient association | Marital status | 0.09 | .77 |  |
|  | Age | 3.52 | .06 |  |
|  | Income | 0.00 | .10 |  |
|  | Educational level | 4.21 | .12 |  |
|  | Type of cancer | 4.66 | .32 |  |
|  | Duration since treatment completion | 4.46 | .22 |  |
| Through Cancer.nl (Kanker.nl) | Marital status | 2.27 | .13 |  |
|  | Age | 0.15 | .70 |  |
|  | Income | 1.24 | .27 |  |
|  | Educational level | 2.35 | .31 |  |
|  | Type of cancer | 1.40 | .84 |  |
|  | Duration since treatment completion | 5.04 | .17 |  |
| Through social media | Marital status | 0.64 | .43 |  |
|  | Age | 0.26 | .61 |  |
|  | Income | 1.26 | .26 |  |
|  | Educational level | 2.51 | .29 |  |
|  | Type of cancer | 4.42 | .35 |  |
|  | Duration since treatment completion | 3.39 | .34 |  |

^1^Adjusted p-values after applying the multiple testing correction method (Benjamin Hochberg FDR correction). This correction is only applied to significant p-values.
*= p-value <.05; **=p-value <.01

Table 3: Results of the binary regression analysis results for predicting answers to the question: “*What factors could prevent you from using digital aftercare programs*?” (Multiple answers possible)

| Answer option | Predictor variable | Wald Chi-Square statistics | P-value | Adjusted p-value^1^ (where applicable) |
| --- | --- | --- | --- | --- |
| I have little energy | Marital status | 0.06 | .81 |  |
|  | Age | 2.82 | .09 |  |
|  | Income | 2.95 | .09 |  |
|  | Educational level | 1.48 | .48 |  |
|  | Type of cancer | 5.17 | .27 |  |
|  | Duration since treatment completion | 0.08 | .99 |  |
| I have difficulty concentrating | Marital status | 0.57 | .45 |  |
|  | Age | 4.78 | .03* | .44 |
|  | Income | 0.01 | .95 |  |
|  | Educational level | 3.13 | .21 |  |
|  | Type of cancer | 7.33 | .12 |  |
|  | Duration since treatment completion | 6.24 | .10 |  |
| Concerns about privacy | Marital status | 0.78 | .38 |  |
|  | Age | 0.69 | .41 |  |
|  | Income | 0.60 | .44 |  |
|  | Educational level | 4.66 | .10 |  |
|  | Type of cancer | 0.50 | .97 |  |
|  | Duration since treatment completion | 3.01 | .39 |  |
| Doubts about the program’s effectiveness | Marital status | 1.44 | .23 |  |
|  | Age | 3.03 | .08 |  |
|  | Income | 0.19 | .66 |  |
|  | Educational level | 4.73 | .09 |  |
|  | Type of cancer | 1.79 | .78 |  |
|  | Duration since treatment completion | 4.62 | .20 |  |
| There are no factors that would prevent me from using digital aftercare programs | Marital status | 3.00 | .08 |  |
|  | Age | 0.47 | .50 |  |
|  | Income | 1.98 | .16 |  |
|  | Educational level | 3.14 | .21 |  |
|  | Type of cancer | 11.41 | .02* | .40 |
|  | Duration since treatment completion | 2.07 | .56 |  |

^1^Adjusted p-values after applying the multiple testing correction method (Benjamin Hochberg FDR correction). This correction is only applied to significant p-values.
*= p <.05; **=p <.01

Table 4: Results of the ordinal regression analysis for predicting answers to the question: “*To what extent do you agree with the following statement: "I would like to address my complaints or challenges independently and online, without the involvement of a healthcare provider or someone else”?*" ((completely) agree, neither agree nor disagree, (completely) disagree)

| Predictor variable | Category | Estimate | S.E. | Wald Chi-Square statistic | P-value | Adjusted p-value (where applicable)^1^ |
| --- | --- | --- | --- | --- | --- | --- |
| Marital status | With partner^2^ | 0.63 | .52 | 1.44 | .23 |  |
| Age |  | 0.03 | .03 | 1.47 | .23 |  |
| Income | Some or significant difficulty^3^ | 0.57 | .53 | 1.15 | .28 |  |
| Educational level | Post-secondary vocational education^4^ | -0.88 | .67 | 1.72 | .19 |  |
|  | Higher professional education or academic education | -0.31 | .62 | 0.25 | .62 |  |
| Type of cancer | Esophageal cancer^5^ | 0.29 | .84 | 0.12 | .78 |  |
|  | Prostate cancer^5^ | -0.19 | .74 | 0.07 | .79 |  |
|  | Bladder cancer^5^ | -0.32 | .60 | 0.28 | .60 |  |
|  | Colorectal cancer^5^ | 0.53 | .58 | 0.83 | .36 |  |
| Duration since treatment completion | Less than one year ago^6^ | -1.32 | .82 | 2.58 | .11 |  |
|  | One to eight years ago^6^ | -1.86 | .71 | 6.82 | .009** | .20 |
|  | Longer than eight years ago^6^ | -1.47 | .98 | 2.25 | .13 |  |

^1^Adjusted p-values after applying the multiple testing correction method (Benjamin Hochberg FDR correction). This correction is only applied to significant p-values; ^2^Reference category (RC) = no difficulty; ^3^RC = without partner ^4^RC = secondary (vocational) education; ^5^RC = breast cancer; ^6^RC = Currently undergoing treatment; *= p <.05; **=p <.01
