# Supplementary material for: What is needed for improved uptake and adoption of digital aftercare programs by cancer survivors: a mixed methods study applying the COM-B model
Source: J Cancer Surviv. 2024 Jul 4;20(1):323–35. doi: 10.1007/s11764-024-01635-x (PMC12906513; doi:10.1007/s11764-024-01635-x)
Supplement: Supplementary file 7 — Supplementary file7 (DOCX 25 KB) [file 11764_2024_1635_MOESM7_ESM.docx]

Supplementary File 7. Interview participants’ characteristics

| **Characteristic** | ***n*** | **(%)** |
| --- | --- | --- |
| *Sex* |  |  |
| - Male | 7 | 50.0 |
| - Female | 7 | 50.0 |
| *Educational level* |  |  |
| - Secondary (vocational) education | 6 | 42.9 |
| - Post-secondary vocational education | 3 | 21.4 |
| - Higher professional education or academic education | 5 | 35.7 |
| *Treatment period* |  |  |
| - Finished treatment <1 year ago | 3 | 21.4 |
| - Finished treatment 1 – 2 years ago | 5 | 35.7 |
| - Finished treatment 3 – 4 years ago | 4 | 28.6 |
| - Finished treatment 5 – 6 years ago | 1 | 7.1 |
| - Finished treatment 7 – 8 years ago | 1 | 7.1 |
| *Cancer type* |  |  |
| - Breast cancer | 4 | 28.6 |
| - Skin cancer | 3 | 21.4 |
| - Bladder cancer | 2 | 14.3 |
| - Colorectal cancer | 1 | 7.1 |
| - Prostate cancer | 1 | 7.1 |
| - Lung cancer | 1 | 7.1 |
| - Lymph node cancer | 1 | 7.1 |
| - Stomach cancer | 1 | 7.1 |
